# Alpha shapes: determining 3D shape complexity across morphologically diverse structures

**DOI:** 10.1186/s12862-018-1305-z

**Published:** 2018-12-05

**Authors:** James D. Gardiner, Julia Behnsen, Charlotte A. Brassey

**Affiliations:** 1 Institute of Ageing and Chronic Disease, University of Liverpool, Liverpool, L7 8TX UK; 2 Manchester X-ray Imaging Facility, University of Manchester, Manchester, M13 9PL UK; 3 School of Science and the Environment, Manchester Metropolitan University, Manchester, M1 5GD UK

## Abstract

**Background:**

Following recent advances in bioimaging, high-resolution 3D models of biological structures are now generated rapidly and at low-cost. To use this data to address evolutionary and ecological questions, an array of tools has been developed to conduct shape analysis and quantify topographic complexity. Here we focus particularly on shape techniques applied to irregular-shaped objects lacking clear homologous landmarks, and propose a new ‘alpha-shapes’ method for quantifying 3D shape complexity.

**Methods:**

We apply alpha-shapes to quantify shape complexity in the mammalian baculum as an example of a morphologically disparate structure. Micro- computed-tomography (μCT) scans of bacula were conducted. Bacula were binarised and converted into point clouds. Following application of a scaling factor to account for absolute size differences, a suite of alpha-shapes was fitted per specimen. An alpha shape is formed from a subcomplex of the Delaunay triangulation of a given set of points, and ranges in refinement from a very coarse mesh (approximating convex hulls) to a very fine fit. ‘Optimal’ alpha was defined as the refinement necessary in order for alpha-shape volume to equal CT voxel volume, and was taken as a metric of overall ‘complexity’.

**Results:**

Our results show that alpha-shapes can be used to quantify interspecific variation in shape ‘complexity’ within biological structures of disparate geometry. The ‘stepped’ nature of alpha curves is informative with regards to the contribution of specific morphological features to overall ‘complexity’. Alpha-shapes agrees with other measures of complexity (dissection index, Dirichlet normal energy) in identifying ursid bacula as having low shape complexity. However, alpha-shapes estimates mustelid bacula as being most complex, contrasting with other shape metrics. 3D fractal dimension is identified as an inappropriate metric of complexity when applied to bacula.

**Conclusions:**

Alpha-shapes is used to calculate ‘optimal’ alpha refinement as a proxy for shape ‘complexity’ without identifying landmarks. The implementation of alpha-shapes is straightforward, and is automated to process large datasets quickly. We interpret alpha-shapes as being particularly sensitive to concavities in surface topology, potentially distinguishing it from other shape complexity metrics. Beyond genital shape, the alpha-shapes technique holds considerable promise for new applications across evolutionary, ecological and palaeoecological disciplines.

## Background

The morphology of an organism is both a function of its evolutionary past and its adaptation to present surroundings. Quantifying morphology is fundamental to the study of ecology and evolution. Organisms are, quite literally, shaped by their evolutionary history. And morphology is often the only source of evidence upon which phylogenetic relationships may be reconstructed through deep time. Morphology also plays important role in linking the phenotype to ecology, by establishing causal relationships between anatomy and performance [1]. Flexible tools for quantifying organismal morphology are therefore highly desirable amongst users spanning the disciplines of ecology and evolutionary biology [2]. More broadly, the comparison of morphological features is of interest to applied scientists from a diverse array of background, including archaeology, chemistry, computer science and medicine.

The morphology of an organism and its component parts can be described in terms of size, shape, structure, colour and patterning. Of these, shape has historically been difficult to consistently and objectively quantify, and this challenge forms the basis of the field of morphometrics. ‘Shape’ can be defined as all the geometric information contained within an object, once the effects of rotation, translation and scale have been removed [3]. Traditionally shape has been quantified as a series of single measurements, including ratios [4] and angles [5]. Such measures clearly ignore a wealth of potential shape data however.

### Shape variation vs. shape complexity

Associated with recent advances in specimen digitization, a suite of new techniques has been developed to analyse 3D biological shape data. Chiefly, these methods facilitate the quantification of variation in form (size and shape) among specimens using multivariate methods, allowing for *either* the study of covariation between shapes *or* between shapes and extrinsic factors [6]. Analyses of biological shape variation most often fall within the paradigm of geometric morphometrics (GMM).

GMM studies typically proceed via the identification of homologous morphological landmarks across a range of specimens, and subsequent Procrustes superimposition to remove the effects of translation, rotation and scale. The placement of landmarks on sutures, muscle attachment scars and tuberosities is therefore common. This approach has proved effective in ecological and evolutionary studies across a range of biological structures, including vertebrate skulls [7], insect wings [8] and tree leaves [9]. More problematic is the landmarking of less featured objects, such as the diaphyses of long bones [10], the body of ribs [11], otoliths [12], seeds [13] and anthropological artefacts [14].

A class of related outline- or surface-based shape analysis tools exist however, that do not necessarily require homologous landmarks to be defined a priori. ‘Eigenshapes’ [15], ‘Eigensurfaces’ [16, 17], ‘Canonical Sampling’ [18, 19], fully automated landmarking (‘auto3Dgm’) [20, 21], ‘Elliptical Fourier Analysis’ [22–25] and ‘Spherical Harmonics’ [26–29] are all important contributions to the morphometricians toolbox of shape analysis techniques. In all such cases, the principle goal of the analysis remains the same: to describe the shape of objects, and the specific ways in which objects differ in shape between themselves and as a function of external factors.

Yet a second suite of morphometric techniques seeks to quantify *shape complexity*. Within the field of biology, complexity may be broadly defined as the number of ‘parts’ comprising an organism or a landscape (be that genes, cell types, organ systems or habitat patches). In the context of shape analysis, here we focus on topographic complexity. Whilst topographic ‘complexity’ has numerous definitions across the literature (see below), complex shapes can intuitively be thought of as those formed by combining parts, or the entirety, of several simple ‘primitive’ shapes. Complexity indices may differ in the specific ‘aspect’ of shape complexity captured, ranging from the degree of self-similarity (fractal architecture) displayed, to simpler metrics of surface rugosity. Shape complexity has found numerous important applications within the disciplines of ecology and evolutionary biology. Root complexity may be indicative of the health of a plant [30], whilst tooth complexity has been used to predict the palaeodiet of fossil vertebrates [31]. The complexity of landscape patches has been linked to habitat quality [32], and invertebrate genital complexity has been interpreted in the context of sexual selection mechanisms [22].

It is important to reiterate that the two suites of morphometric techniques highlighted above measure two very different aspects of form (namely shape variation vs. complexity), such that two objects may occupy very similar GMM morphospace whilst being characterised by different values of shape complexity. Two outwardly similar surface meshes with similar landmark configurations may differ in shape complexity if the surfaces deviate in terms of surface rugosity, for example.

### Methods for quantifying shape complexity

Several metrics have been advanced for the quantification of spatial or topographic complexity within biological systems:

#### Dissection index and relief index

In two dimensions, dissection index (DI) is the ratio of an object’s perimeter to the square root of its area. Dissection index is therefore a dimensionless number, providing an indication of the extent to which a shape is more complex than a circle [33]. In three dimensions, the related Relief Index (RFI) is calculated as the ratio of an object’s surface area to its planar area [34], and thus provides an index of rugosity. Both metrics are simple to calculate and intuitive to understand, and represent single-parameter shape descriptors of complexity. A corollary of this however, is neither metric provides an indication of the *distribution* of complexity across an object. Furthermore, the value of planar area incorporated into RFI is necessarily orientation-dependent (total planar area is dependent upon on the orientation of the object relative to the observer when the plan view is taken). When applied to tooth complexity, the preferred orientation is obvious; planar area is calculated in the occlusal plane when RFI is taken as a proxy for hysodonty [34]. Should RFI be extended beyond tooth crown complexity to other biological structures however, the orientation of planar area will need further consideration. Additionally, the calculation of RFI requires a mesh from which to derive surface area, involving an intermediate processing stage for point clouds or voxel- based data.

#### Fractal dimension

The fractal dimension (FD) is a measure of complexity applicable to objects that are self-similar (exhibiting repetitive patterns across scales) [35]. FD metrics have commonly been applied to physical landscapes [36] in addition to biological organisms perceived to display self-similarity, including plant roots [37], plant leaves [38], stony corals [39], and brain structures [40]. Simply speaking, the fractal dimension captures the ability of an object to fill the Euclidean space within which it is located. The most common implementation of FD applies a ‘box-counting’ approach, in which a regular grid of boxes of side length *s* is overlain across the 2D data and the number of occupied boxes counted as *N(s)*. This process is repeated whilst varying the size of *s*. log *N(s)* is subsequently plotted as a function of log (1/*s*), and the slope of this graph is taken as an estimate of FD [41]. Whilst originally implemented on 2D data, fractal analysis has since been extended to operate in 3D [39, 40].

Fractal analysis is undoubtedly a powerful tool that provides an objective and scale-independent single metric of shape complexity. However, numerous caveats have been expressed when applying FD to biological datasets (see [41] for a review). Most notably, when an object or pattern is not obviously self-similar, the application of fractal dimensions can be problematic [42]. Indeed, rather than being truly self-similarity, some authors have gone so far as to suggest that most ‘complex’ structures differ in their extent of self-similarity across *spatiotemporal* scales, and are actually best described as self-dissimilar [43]. Furthermore, the value of the fractal dimension for a given outline is a function of several ‘somewhat arbitrary’ decisions, including the location of the grid starting point and the selected values of minimum and maximum *s* [44]. Within the ecological literature, occupancy is typically calculated across *s* values spanning ~two orders of magnitude, yet such a limited scaling relationship cannot be taken as strong evidence of genuine fractality [41].

#### Dirichlet Normal energy

Dirichlet normal energy (DNE) effectively quantifies the ‘curviness’ of a mesh. Most simply “DNE measures the deviation of a surface from being planar” ([45]; p249), and ranges from zero in the case of a flat plane, to higher values associated with steep crests and troughs. It is calculated as the sum of energy values across all faces of a mesh surface, where the energy value at each face is quantified as changes in the normal map. The process does not require the assignment of landmarks, and is unaffected by scale or orientation. Additionally, energy is calculated for every face of the mesh, facilitating energy variation to be visualized across the surface of the object. In this way, DNE allows for specific regions of ‘high’ and ‘low’ complexity to be identified across a specimen. Thus far, DNE has found extensive use in the mammal tooth literature [45–47], but has also been applied to quantify the shape complexity of developing embryos [48]. DNE does however, require a mesh a priori, and has been shown to be sensitive to commonly-used mesh preprocessing operations such as smoothing and decimating [49].

### Alpha-shapes

In this study, our objective is to develop a straightforward method for quantifying three-dimensional shape complexity that is orientation-independent, does not require assumptions of self-similarity or an intermediate meshing stage, and is capable of quantify topographic complexity across multiple scales. Our approach is based on the concept of ‘*alpha-shapes’*.

**Fig. 1:**
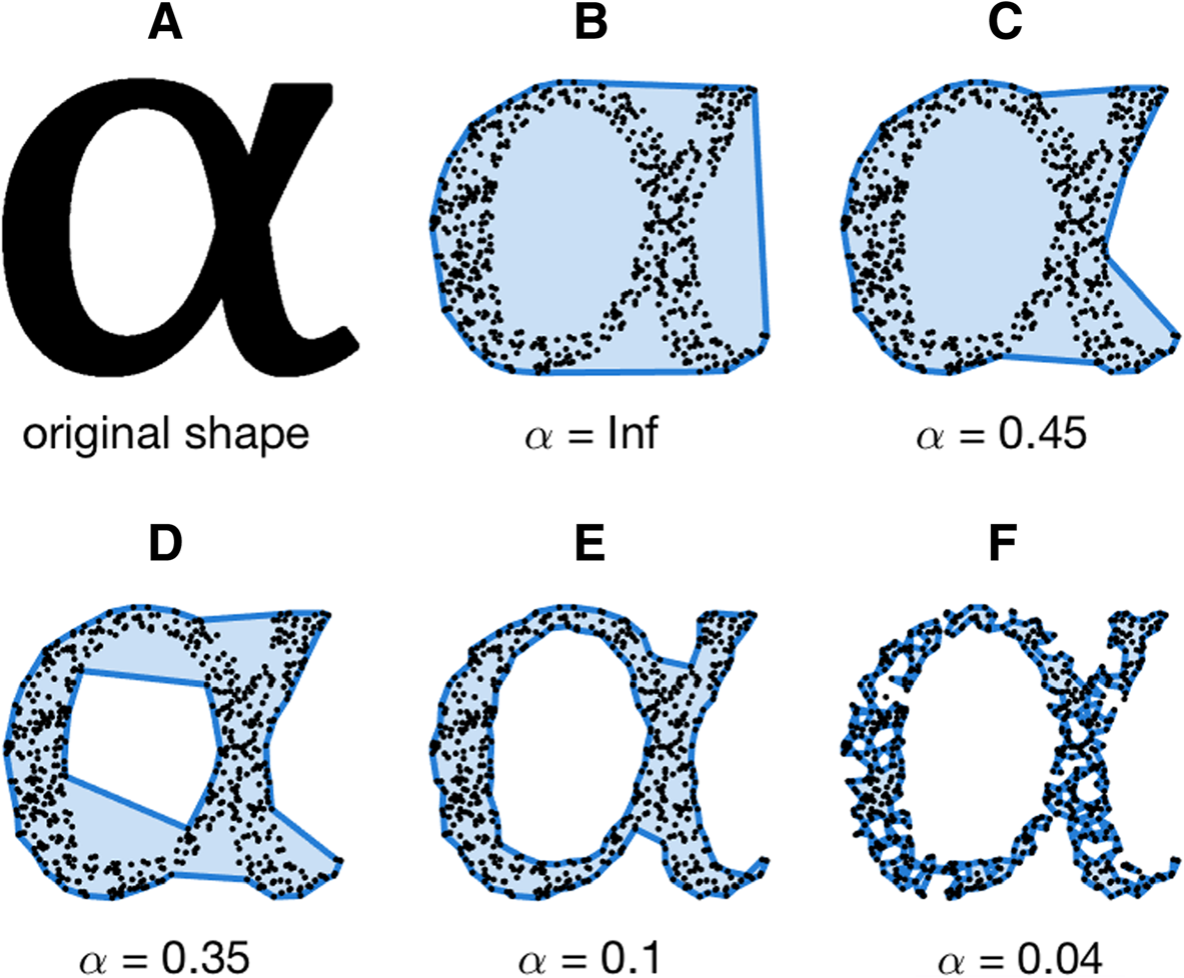
Diagram illustrating the nature of alpha-shapes, as understood in 2-dimensions. **a**, the original shape to which alpha-shapes are to be fitted; **b**, a convex hull fitted to the data representing the special case when alpha = infinity; **c**-**e**, represent increasingly refined alpha-shapes fitted to the data as alpha is reduced; **f**, represents the point at which the alpha radii can pass ‘internally’ through the data set and the alpha-shape breaks down to form several smaller shapes. Intuitively, the alpha-shape represented in Fig. 1e would be considered as ‘most-representative’ of the original shape described in Fig. 1a, as defined by equivalency of area

An alpha-shape is formed from the boundary of an alpha-complex, which is itself a subcomplex of the Delaunay triangulation for a given set of points [50]. For a given set of points in space, a family of alpha-shapes may be defined, ranging from a very coarse (a convex hull) to very fine fit around said points (Fig. 1). The parameter ‘alpha’ dictates the level of refinement, with a larger alpha resulting in coarser fits and a smaller alpha in finer fits (see [50] for a comprehensive description of 3-dimensional alpha-shapes). The level of refinement necessary in order for an alpha-shape’s volume to match that of the original dataset to which it is fitted may be taken as a measure of shape complexity: more complex objects will require a more refined alpha-shape fit in order for volumes to converge.

The resulting alpha-shape fit may comprise one volume (larger alphas; Fig. 1c-e) or multiple smaller volumes (smaller alphas; Fig. 1f). Hence, as alpha decreases, the refinement of the fit changes from a convex hull (the special case when sphere radius is infinite, Fig. 1b) to finer fits as more regions are removed by smaller spheres (Fig. 1c-e). Eventually the radius of the sphere decreases to such an extent that no points are intersected and no alpha-shape is created.

The convex hull (Fig. 1b) and ‘coarser’ alpha-shapes (Fig. 1c-d) occupy a volume *equal to- or larger* than that of the underlying object (Fig. 1a). In contrast, very fine alpha-shapes (Fig. 1f) will have a volume *smaller* than the original structure. At some ‘optimal’ level of refinement, alpha-shape volume and specimen volume will be equal (Fig. 1e), and it is this ‘optimal’ alpha upon which we base our metric of 3D shape complexity.

Within the biological sciences, alpha-shapes have previously been used to describe characteristics of protein surface shape [51], to segment forested areas from aerial LiDAR data [52] and to describe the spatial distribution of fish within schools [53]. In a practical sense, alpha-shapes has been implemented in the freeware ‘Meshlab’ [54] as a means of generating surface meshes from point cloud data. The authors have previously applied an alpha-shapes approach to the problem of body mass estimation in fossil species [55]. In this implementation, a predictive relationship between alpha shape volume and body mass was derived from a suite of articulated modern mammal skeletons digitised using LiDAR. The predictive model was subsequently applied to extinct mammal taxa and their fossil body mass estimated. To the authors knowledge, alpha-shapes has not previously been applied to explicitly quantify shape complexity however.

### Genital shape complexity

Within the field of evolutionary biology, genital form and function has received considerable attention, albeit with a heavy bias towards invertebrates [56]. Genitals are amongst the most diverse, complex and rapidly evolving structures observed in living organisms [57]. Genital shape, rather than size, is often used by taxonomists as a means of distinguishing between closely related species [58, 59], implying greater divergence in genitalic shape than size [22]. Indeed, numerous experimental evolution studies have found direct evidence for sexual selection acting on genital shape across a range of taxa [23, 60, 61].

There is therefore considerable interest in developing automated methods capable of quantifying shape across such complex and diverse structures as animal genitalia. In some instances, traditional landmark-based GMM techniques have been applied [60, 62–65]. Such studies frequently consider genital shape variation *intraspecifically,* or between morphologically similar sister taxa [66, 67]. Yet elsewhere, GMM methods have been applied to broader *interspecific* samples of genitalia [65, 68, 69], highlighting the applicability of these techniques to quantify shape change in rapidly evolving structures, or those comprised entirely/predominantly of soft tissue [70].

Here we use the mammalian baculum as a test case for the application of alpha-shapes to qualifying morphological complexity. In the past, bacula have been used as a taxonomic character to differentiate between otherwise indistinguishable sister taxa, such is their morphological disparity between closely related species. Whilst this is predominantly true for rodents and bats [71], baculum morphometrics have also been developed as a diagnostic tool for differentiating between species of carnivore [72]. As far as the authors are aware, a traditional geometric morphometric analysis of baculum shape has not been attempted *between* species however, potentially due to difficulties associated with the identification of discrete homologous landmarks. The development of a simple and intuitive method for quantifying ‘complexity’ in the mammal baculum in the absence of homologous landmarks therefore has the potential to reinvigorate the study of mammal genital evolution. In addition, the present study is of significance for both ecologists and evolutionary biologists (and those working more broadly in the fields of archaeology and computer science) who will benefit from a new tool for comparing 3D shape complexity across samples of extreme shape diversity.

## Methods

### Raw data

**Fig. 2:**
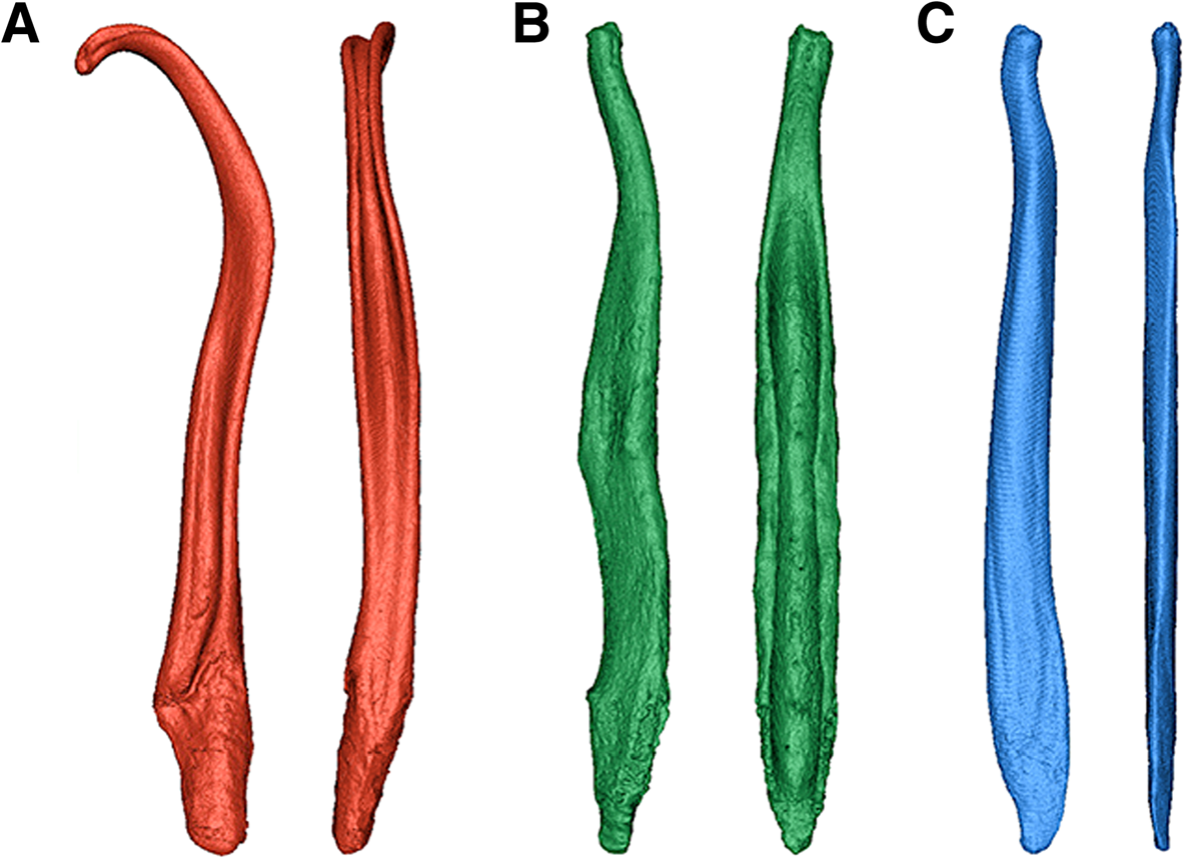
Surface renderings (lateral and ventral view) of three example Carnivora bacula, for illustrative purposes. **a**. *Mustela kathiah*; ***b****. canis lupus*; ***c****. ursus maritimus*

Twelve mammalian bacula were scanned as an example dataset using micro- computed tomography (μCT). The taxa include three families of modern Carnivora (Mustelidae, Canidae and Ursidae; Table 1) and span a range of shapes (Fig. 2) from simple rod-like bones (Ursidae) to complex curved, grooved and notched structures (Mustelidae).

**Table 1:** Baculum specimens included in analysis

Family	Taxa	Common name	Accession number	Baculum length (mm)	Scan resolution (mm)	Voltage (kV)	Current (uA)	Filter (mm)
Mustelidae	*Mustela itatsi*	Japanese weasel	84.2.9.1^a^	30.4	0.031	150	160	Cu 0.1
Mustelidae	*Mustela kathiah*	Yellow-bellied weasel	33.4.1.248^a^	29.9	0.023	150	160	Cu 0.1
Mustelidae	*Mustela lutreola*	European mink	PH133.06^b^	35.3	0.032	75	80	NA
Mustelidae	*Mustela nigripes*	Black-footed ferret	Z.1999.206.003^b^	29.9	0.032	75	80	NA
Ursidae	*Melursus ursinus*	Sloth bear	Z.2001.42.2^b^	156.6	0.050	100	90	Cu 0.1
Ursidae	*Tremarctos ornatus*	Spectacled bear	Z.2001.42.2^b^	140.1	0.050	100	90	Cu 0.1
Ursidae	*Ursus arctos*	Brown bear	1938.6.24.3^a^	122.8	0.067	140	150	Cu 0.1
Ursidae	*Ursus maritimus*	Polar bear	Z.2000.234^b^	186.8	0.050	100	90	Cu 0.1
Canidae	*Canis aureus*	Golden jackal	5.10.4.18^a^	64.1	0.048	140	150	Cu 0.1
Canidae	*Canis lupus*	Grey wolf	LW3^b^	99.9	0.040	75	80	NA
Canidae	*Canis mesomelas*	Black-backed jackal	820^a^	52.6	0.031	140	150	Cu 0.1
Canidae	*Chrysocyon brachyurus*	Maned wolf	Z.200.27^b^	97.7	0.040	75	80	NA

^a^Natural History Museum, London. ^b^National Museum of Scotland, Edinburgh

CT scans were conducted at Manchester X-ray Imaging Facility using a Nikon 320/225 kV Custom Bay microCT instrument, and the Natural History Museum London using a Nikon 225 kV microCT instrument. Raw CT scans were converted to binary data in ImageJ by automated thresholding according the histogram of raw CT grayscale values. Binarised CT scans were read into MATLAB R2017a (The MathWorks Inc., Natick, MA, USA) slice by slice, and any internal cavities present were filled using two separate automatic gap and hole filling algorithms, (imclose.m and imfill.m) from MATLAB’s Image Processing toolbox. Imclose.m performs a morphological closing on each binary image slice, using a 2D disc of a given radius. In this instance, 6 pixels was found to be the minimum radius that consistently closed the periosteal contour across the sample. Imfill.m identifies holes as being background pixels that cannot be reached from the edge, and subsequently flood fills them with foreground pixels. The relative ‘hollowness’ of bacula has not previously been described, yet all specimens included in the present study did possess internal void spaces. Here we chose to focus on the shape complexity of the external morphology, and hence filled any internal cavities. Nevertheless, the alpha-shapes technique will function equally well for instances when the internal geometry is pertinent to the research question.

Having filled internal void spaces, CT data were converted directly to point clouds. This highlights an important advantage of the alpha-shapes approach as a means of directly calculating shape ‘complexity’ from a CT dataset via the *process* of surface meshing, rather than requiring a surface mesh beforehand. The process of converting CT volumes to surface meshes necessarily involves some degree of smoothing to avoid faceting and topological artefacts resulting from image artefacts and noise. This process ought to be, but is rarely, documented in the metadata [73] and the effect of smoothing on subsequent data analysis is seldom explored. Raw point clouds were generated by designating the x-y-z coordinates of every voxel in the CT segmentation associated with the baculum as being a single point in space. That is, unlike surface-based point clouds generated by other popular digitisation techniques such as LiDAR (light detection and ranging) or photogrammetry, here point clouds also comprise ‘internal’ points representing the solid infilled bone. Raw point clouds were randomly downsampled to 100,000 points each, ensuring all specimens were represented by equally sized datasets (but see ‘Sensitivity Analysis’ below).

### Alpha-shapes

#### Alpha and reference length

Prior to fitting alpha-shapes, the issue of scale must be dealt with. Here we are interested in quantifying shape ‘complexity’ in the absence of potential size signals. Alpha radii are calculated in the same units as the underlying point clouds, therefore an alpha radius of 100 mm may entirely enclose one smaller specimen yet only half of another larger specimen, for example. Size normalisation may be achieved in one of two ways: *either* by scaling all point clouds to the same size, *or* by scaling alpha radii to the overall size of each specimen.

In this implementation of alpha-shapes, we choose the later. In doing so, the underlying point cloud remains the same scale throughout. A specimen with a maximum length of 100 mm will remain represented by a ~ 100 mm-long point cloud. The resulting alpha shapes mesh, comprising all the triangles formed when the points contributing to the alpha shape are connected, will likewise remain at this original scale and may then be used in downstream functional analyses (see Discussion). Therefore, if two objects are identical in shape and both comprise an equal number of points, yet one is twice the size, the larger specimen requires an alpha radius (*α*) twice as large to ensure an equal refinement of fit. To calculate the alpha radius for each specimen we use the following equation:

1 $$ \alpha =k\ast {l}_{ref} $$

where *α* is the alpha radii, *k* is the refinement coefficient and *l*_*ref*_ is the point cloud reference length (as described in the following section). Here we are interested in identifying an ‘optimal’ level of alpha-shape refinement (see below) and therefore chose 200 values of refinement coefficient *k* that result in alpha-shapes ranging from coarse fits (convex hulls) to highly refined shapes. Refinement coefficients ranged from 0.1 to 10,000 and were evenly distributed on a logarithmic scale. At the smallest values of refinement, the alpha-shape ceases to be one continuous volume and the sphere passes inside the point cloud to create multiple small volumes, hence no longer representing the overall shape of the object.

#### Scaling reference length

**Fig. 3:**
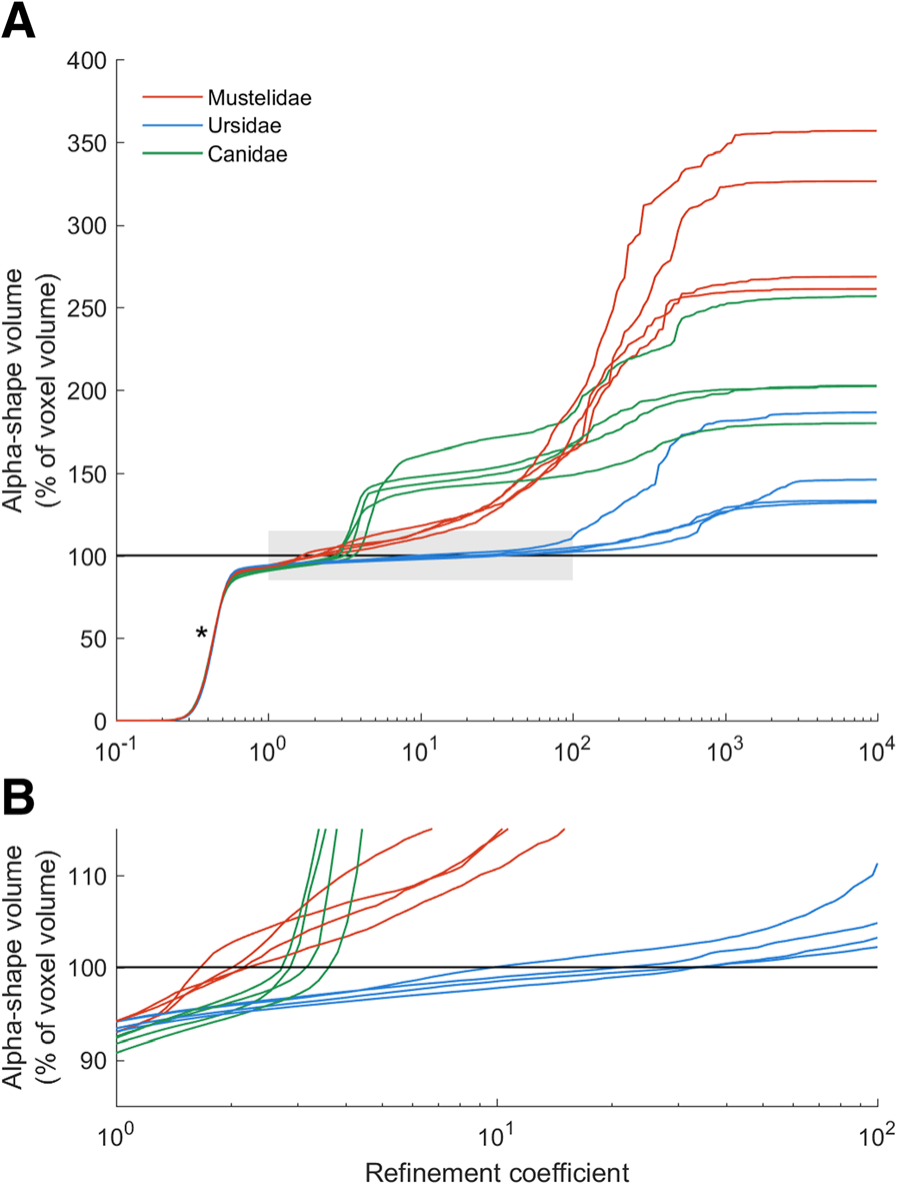
Alpha-shapes results for all specimens. **a**, bacula of outwardly ‘similar’ shape complexity describe similar alpha-shape curves; **b**, zoomed-in grey region of Fig. 3a. The location at which alpha volume crosses 100% of CT voxel volume is taken at the ‘optimal’ refinement coefficient and used as a metric of overall shape complexity. Mustelids require small values of refinement coefficient to adequately represent their geometry, whereas comparatively ‘simple’ ursid bacula can be described by coarser alpha fits. The point at which all alpha-shapes break down into multiple smaller volumes (*) is consistent for all specimens, suggesting that alpha radii is well scaled to the overall size of the point cloud

The point cloud reference length *l*_*ref*_ is a scaling factor allowing equivalencies to be drawn between alpha-shapes fitted to specimens of absolute different size, as discussed above. Yet arriving at a single ‘reference’ length that adequately describes the overall size of a point cloud is non-trivial. A simple approach is to use the maximum diagonal of the bounding box as a reference length. Alternatively, an earlier implementation of alpha-shapes as a mass estimation tool [55] utilised the average distance of all points from the centroid of the point cloud. Here, we investigate a third technique, in which the average distance of each point to its nearest 100 neighbours in the downsampled point cloud is used as a descriptor of overall point cloud size. Ultimately, the nearest neighbour technique was preferred as this resulted in alpha-shapes ‘breaking down’ (i.e. becoming multiple small volumes) at the same refinement coefficient (Fig. 3 - see asterisk *), implying alpha radii is well scaled to the relative distance between points in the point cloud, and therefore the overall size of the specimen.

#### Optimal refinement coefficient

Having calculated alpha radii using the above equation, alpha-shapes were fitted to point clouds using the MATLAB ‘alphavol’ function written by Jonas Lundgren (http://www.mathworks.co.uk/matlabcentral/fileexchange/28851-alpha-shapes), which both calculates the fit of the alpha-shape and its associated volume. Alpha-shapes were fitted for a range of refinement coefficient across all specimens, and volumes extracted. All analyses were run on a laptop computer with 8GB 1600MHz DDR3 RAM and a 1.1GHz Intel Core M processor.

Each specimen is described by a representative curve of alpha-shape volume against refinement coefficient. As alpha-shapes become more refined (smaller refinement coefficients), their associated volumes decrease. However, the profile of this alpha curve is a function of the shape complexity of the bone, from gross overall shape (straight vs curved, for example), to specific morphological features (such as grooves or forked tips) and ultimately surface texture/roughness (i.e. pitted or smooth). The alpha curve often has a stepped appearance, with steep regions corresponding to a sudden reduction in alpha volume when alpha radius becomes sufficiently small so as to represent a particular feature or surface texture.

For each specimen, we therefore identify the ‘optimal’ refinement coefficient best reflecting overall shape complexity by comparing alpha volume against ‘raw’ volume. ‘Raw’ volume is an estimate of the biological volume of the specimen, as calculated from the hole filled CT data by multiplying the number of threshold voxels by scan resolution cubed, prior to point cloud downsampling. The refinement coefficient producing an alpha volume that is closest to raw volume will be taken as the ‘optimal’ refinement. To identify the ‘optimal’ refinement coefficient an optimisation approach was undertaken using the ‘fminsearch’ function of MATLAB’s optimisation toolbox, which applies a ‘Nelder-Mead’ search method. The optimisation routine searches for the refinement coefficient that produces the smallest difference between alpha volume and raw volume. This process continues until two conditions have been satisfied: the difference between volumes (alpha volume vs raw volume) is less than 1e-4, *and* the difference between subsequent values of refinement coefficient is less the 1e-4. The final refinement coefficient after both conditions have been satisfied is taken as ‘optimal’.

Using our approach, 3D shape complexity is reduced first into one curve per specimen and ultimately into one refinement value per specimen (see supplementary material for MATLAB code). We predict that simple rod-like structures will require a relatively coarser refinement coefficient (relatively larger alpha radii) to match ‘raw’ volumes compared to complex, curved or grooved specimens that will require a more refined alpha shapes (relatively smaller alpha radii) to accurately represent total volume.

#### Comparison to other shape complexity measures

Here we compare alpha-shapes to three additional metrics of topographic complexity commonly applied in the field of evolutionary biology. Firstly, we calculate the orientation-independent 3D ‘dissection index’ (DI) which represents the ratio of the squared root of surface area to the cubed root of volume. 2D dissection indices have previously been applied to quantify shape complexity in invertebrate genitalia [22], and here we modify this technique to work on 3-dimensional data. Isosurface meshes (comprising 10,000 faces, see DNE section below) were generated from the binarised .raw CT stack in Horos [74], decimated in Geomagic (3D Systems, North Carolina, USA) and surface areas and volumes calculated using the ‘compute geometric measures’ function in Meshlab [54].

Three-dimensional fractal dimension (FD) was estimated using a box-counting algorithm written in MATLAB by Frederic Moisy (https://uk.mathworks.com/matlabcentral/fileexchange/13063-boxcount). The function was applied to the binarized CT data after the internal cavities present were filled, but prior to conversion to a point cloud and downsampling (see ‘Raw data’ section above). The function calculates the number of cubes required to cover the baculum *N(s)* at sequential sizes of box, where the size of the cube *s* is the length of one side. The slope of the relationship of log(1/*s*) to log *N(s)* is taken as an estimate of the FD of the object, where objects with higher topographic complexity have a higher values FD.

Dirichlet Normal Energy (DNE) was calculated in the R package ‘MolaR’ following the methodology of Pampush et al. [47], using the same surface meshes as produced for the 3D DI calculation above. As per previous applications, meshes comprised 10,000 faces [47]. Higher values of DNE are indicative of higher topographic complexity.

#### Sensitivity analysis

In theory, alpha shapes can cope with infinitely detailed point clouds, yet practically the number of points comprising an object will be dictated by several factors. The scanning technique used can impact the density of the point cloud, with μCT scans often producing very dense point clouds compared to LiDAR or photogrammetry (although this does strongly depend upon the specifics of a given imaging set-up). Larger point clouds necessitate longer computational times, which may problematic for large comparative studies. More importantly, the particular research question ought to have a large bearing on the density of the point cloud. If the question under investigation pertains to ‘gross’ morphology, a less dense point cloud may be justifiable, whereas those focusing on features of surface texture may require more detail. Whilst final point cloud size is ultimately determined by the users’ needs, ensuring that all specimens within a comparative study comprise an equal number of points is necessary in order that one sample is not represented in significantly more detail than another, which may potentially skew the results.

We therefore conducted a sensitivity analysis to examine the effect of point cloud density on calculated values of ‘optimal’ refinement coefficients (and associated computing time). Optimal refinement coefficients were calculated for each specimen comprising points cloud sizes ranging from 10^4^ to 10^6^ points, typical for datasets derived from LiDAR, photogrammetry or CT. Reference lengths (see Eq. 1) of the 10^4^ point clouds were used to scale alpha radii for all point cloud sizes, ensuring consistent alpha radii (at each refinement) between point cloud sizes and that results are equivalent.

## Results

The alpha-shapes methodology described here distils the complexity of three dimensional baculum shape, firstly into a single representative curve and ultimately into a single parameter to facilitate further comparative analysis. We consider the shape-fitting protocol to be straightforward and relatively computationally inexpensive when operating on point clouds of ~ 100,000 data points. For a typical specimen (*Mustela itatsi,*14 MB 8 bit raw file), data import and hole filling took 14 s, the calculation of reference length on the basis of 100 nearest neighbours took 14 min, and the calculation of the optimal refinement coefficient took 2 min.

**Fig. 4:**
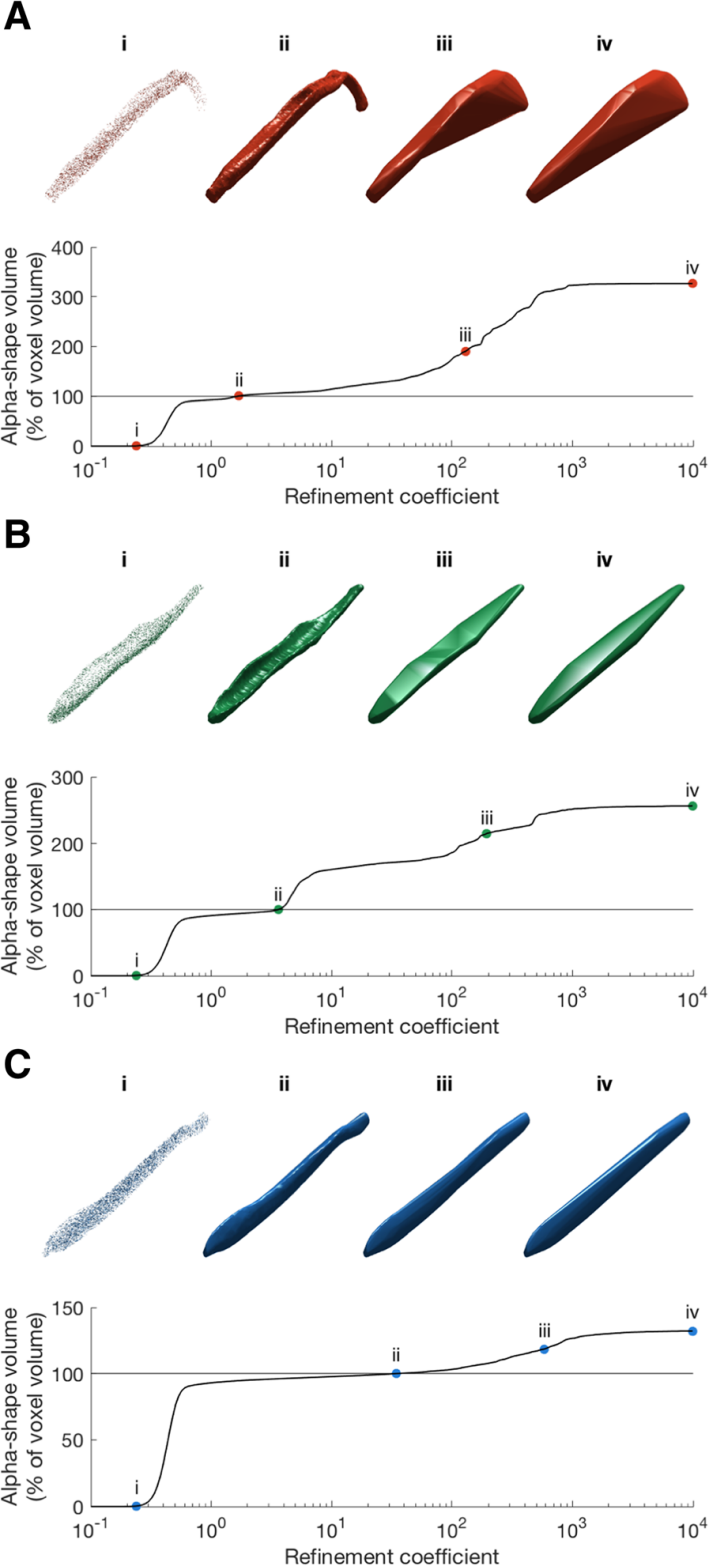
Alpha-shapes fitted to three example bacula. **a**, mustelid; **b**, canid; **c**, ursid. As refinement coefficient is decreased, the volume of alpha-shapes (as a percentage of CT voxel volume) decreases. i, when this value drops below 100, the alpha-shape has ‘broken down’ and the fit passes internally of the point cloud; ii, the ‘optimal’ refinement occurs when alpha volume is exactly equal to CT volume; iii, an intermediate fit alpha-shape defined as halfway between ‘optimal’ alpha and the cnvex hull describes some coarser geometric features, such as the curvature of the mustelid baculum, but misses finer-scale detail such as the canid urethral groove; iv, the coarsest alpha-shapes are equivalent to convex hulls, fitted only to the outermost extremes of the point cloud and representing gross morphology. Due to the curved nature of the mustelid baculum, coarse alpha-volume is considerably greater than the CT voxel volume

In specimens appearing outwardly similar, the relationship between alpha-shape volume and refinement coefficient is characterised by similar profiles. Ursid bacula, for example, share a simple rod-like appearance which is smooth and lacking in features such as grooves, curvature or complex apices (Fig. 2 and Fig. 4), and likewise the four bear bacula share similar alpha curves (Fig. 3). At the highest value refinement coefficients (approaching a convex hull), ursid alpha-shapes overestimate raw volume by ~ 25–50%, and only the outermost points of the point cloud contribute to shape fitting. As refinement coefficients decrease, divergence between alpha-shape volume and raw volume is quickly reduced, and ‘optimal’ alpha is reached (occurring at refinement coefficients between 11 and 36; Table 2). Beyond which, alpha-shape volume decreases with refinement coefficient at a slower rate, until the alpha-shape fit breaks down to form several disconnected volumes (refinement coefficients below 0.6).

**Table 2:** Optimal values of alpha derived from shape-fitting protocol, compared to 3D dissection index values, fractal dimensions and Dirichlet normal energies

Family	Taxa	Optimal refinement coefficient	Dissection index (^2^√SA/^3^√V)	Fractal dimension	Dirichlet normal energy
Mustelidae	*Mustela itatsi*	1.67	3.54	2.17	1241
Mustelidae	*Mustela kathiah*	2.04	3.57	2.16	1427
Mustelidae	*Mustela lutreola*	2.36	3.3	2.24	1330
Mustelidae	*Mustela nigripes*	2.18	3.3	2.26	1384
Ursidae	*Melursus ursinus*	20.9	3.09	2.24	656
Ursidae	*Tremarctos ornatus*	10.7	3.12	2.20	578
Ursidae	*Ursus arctos*	32.5	3.29	2.11	826
Ursidae	*Ursus maritimus*	36.2	3.37	2.10	718
Canidae	*Canis aureus*	2.87	3.87	2.13	1242
Canidae	*Canis lupus*	3.18	3.81	2.20	1389
Canidae	*Canis mesomelas*	3.59	4.1	2.12	1460
Canidae	*Chrysocyon brachyurus*	2.71	3.9	2.11	1714

SA, surface area; V, volume

**Fig. 5:**
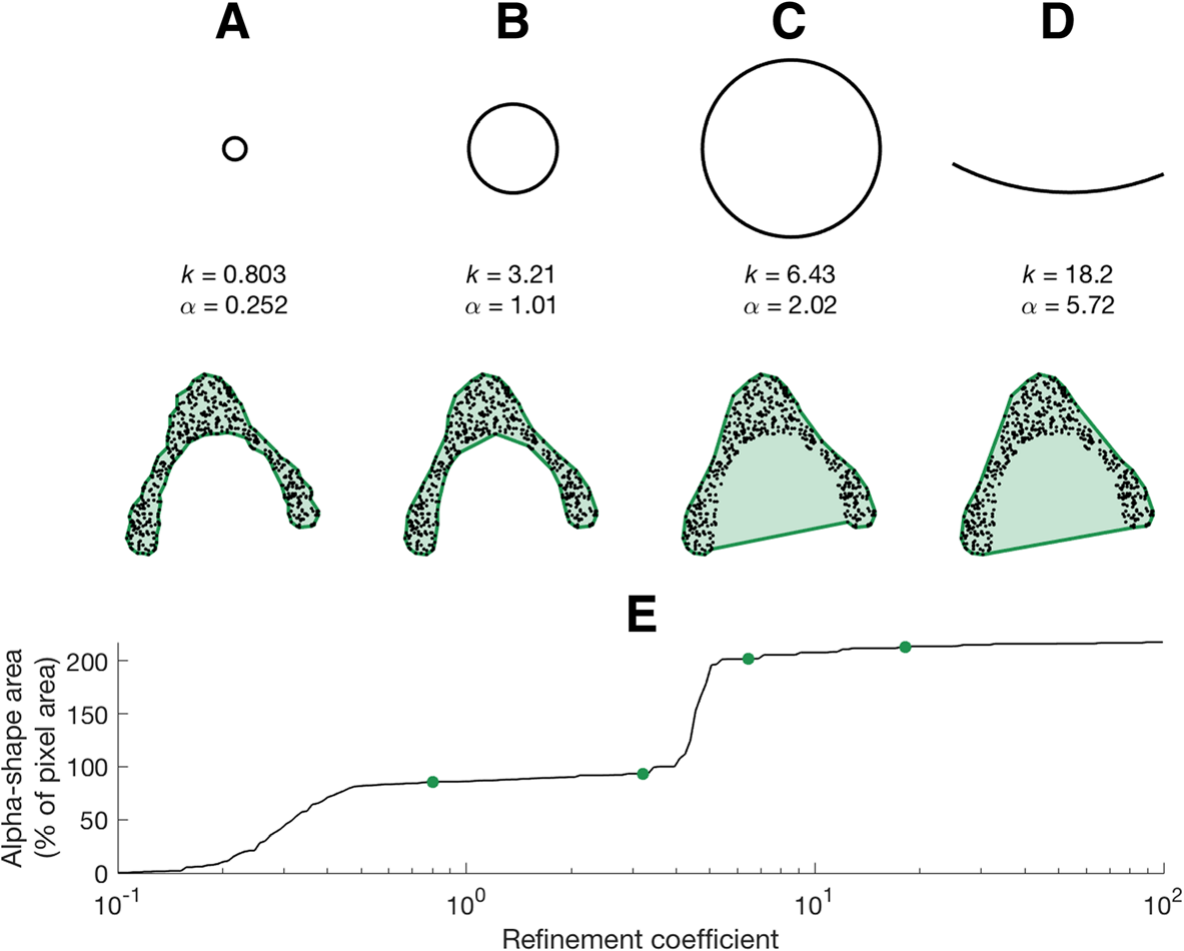
The stepped alpha-shape profile of a canid baculum (modelled in 2-dimensions for illustrative purposes). Circles illustrate the value of alpha radius at four locations (**a**-**d**) along the alpha curve. The step between **b** and **c** represents the point at which the alpha exceeds the width of the urethral groove. Once the groove is no longer distinguished, alpha volume increases dramatically

Whilst also lacking distinct curvature or a complex tip, the canid baculum does possess a well-developed broad urethral groove on the ventral surface (Fig. 2 and Fig. 4). Optimal refinement coefficients of canid bacula are therefore intermediate between those of ursids and mustelids (Table 2). It follows that these specimens have a more complex relationship between alpha-shape volume and refinement coefficient, with curves taking on a multi-stepped appearance (Fig. 3). Steps coincide with refinement coefficient values becoming small enough to allow specific morphological features to be detailed. At high values of refinement, alpha-shapes overestimate canid baculum raw volume by ~ 200%. As the refinement coefficient is reduced, canids display a very pronounced ‘step’ (Fig. 3 iii to ii) at a refinement coefficient of ~ 5. This coincides with alpha radii falling below ~half urethral groove width (Fig. 5) and the distinctive feature suddenly being resolved. Optimal alpha occurs soon after at refinement coefficients of 2.7–3.6.

Finally, mustelids require low values of refinement coefficient to accurately represent raw volume, as expected due to their complex geometry. In *Mustela itatsi* (Fig. 4a) for example, alpha-shape volumes generated by high values of refinement coefficient vastly exceed raw volume (by a factor of ~ 3), due to the highly-curved nature of the bone. As refinement coefficient is reduced, previously unseen morphological features become apparent. Overall dorsoventral curvature is defined at a refinement of ~ 10 (Fig. 4a iii) whereas more detailed morphological features such as the urethral groove and the rugose proximal portion associated with attachment to the corpora cavernosa become apparent at a refinement of 1.7 (Fig. 4a ii). Due to the overall complex shape of this structure, alpha-shape volume converges upon raw volume to produce an ‘optimal’ refinement fit at low values of refinement.

**Fig. 6:**
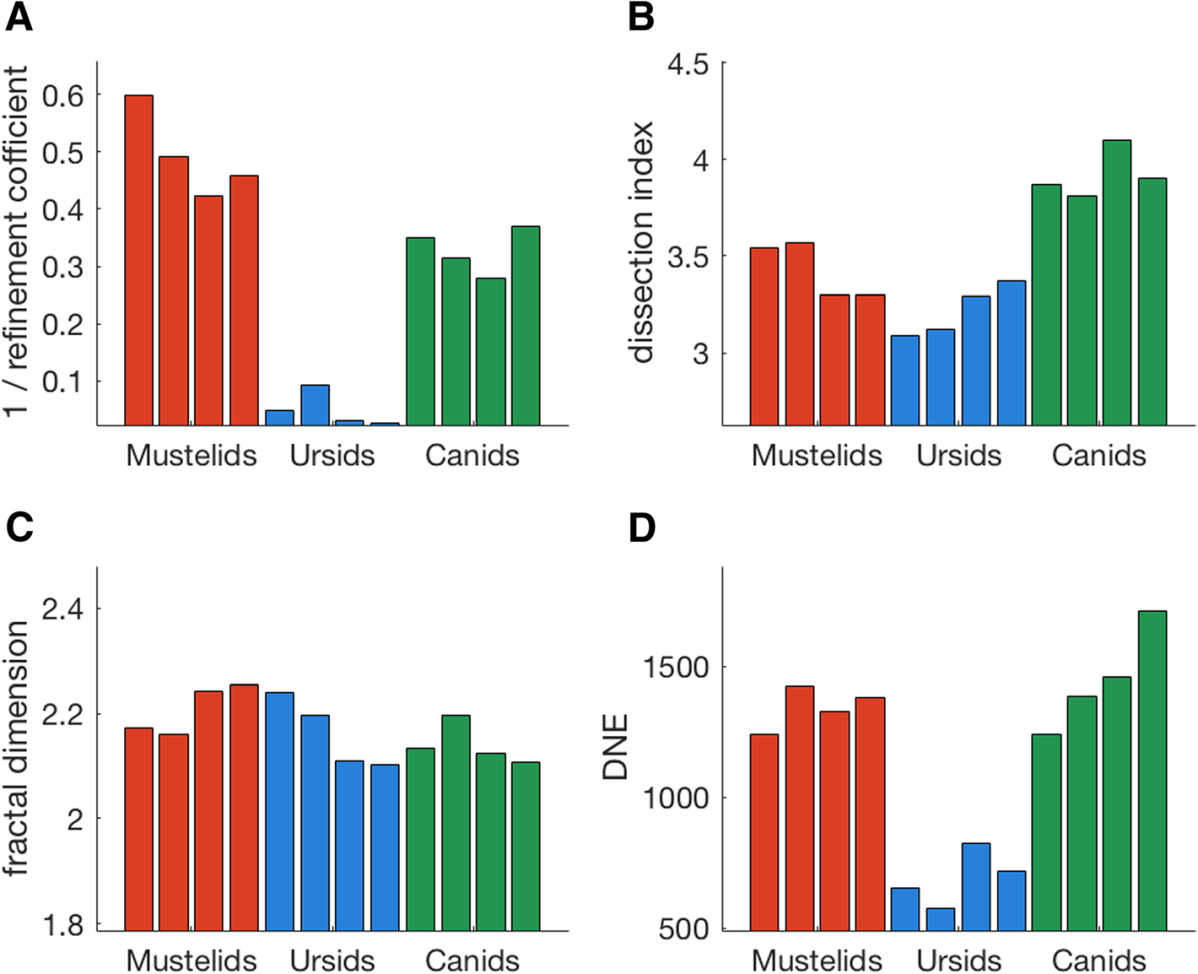
A comparison of four metrics for quantifying topographic shape complexity, as applied to carnivore bacula. **a**, alpha-shapes (displays 1/refinement coefficient, such that lower values indicate less complex shapes, in line with other metrics); **b**, dissection index (DI); **c**, fractal dimension (FD) estimated using box-counting; **d**, Dirichlet normal energy (DNE) calculated from surface mesh

Both DI and DNE complexity metrics agree with the alpha shapes methodology presented here in finding ursids to possess low complexity bacula (Table 2, Fig. 6). Alpha-shapes is the only metric tested here in which all taxonomic groups are entirely differentiated from each other on the basis of surface complexity. In contrast, there is very little differentiation between family groupings when baculum complexity is quantified by fractal dimension (Table 2, Fig. 6). Average DI and DNE values of canid bacula exceed those of mustelids, reversing the trend present in optimal alpha.

### Sensitivity analysis

**Fig. 7:**
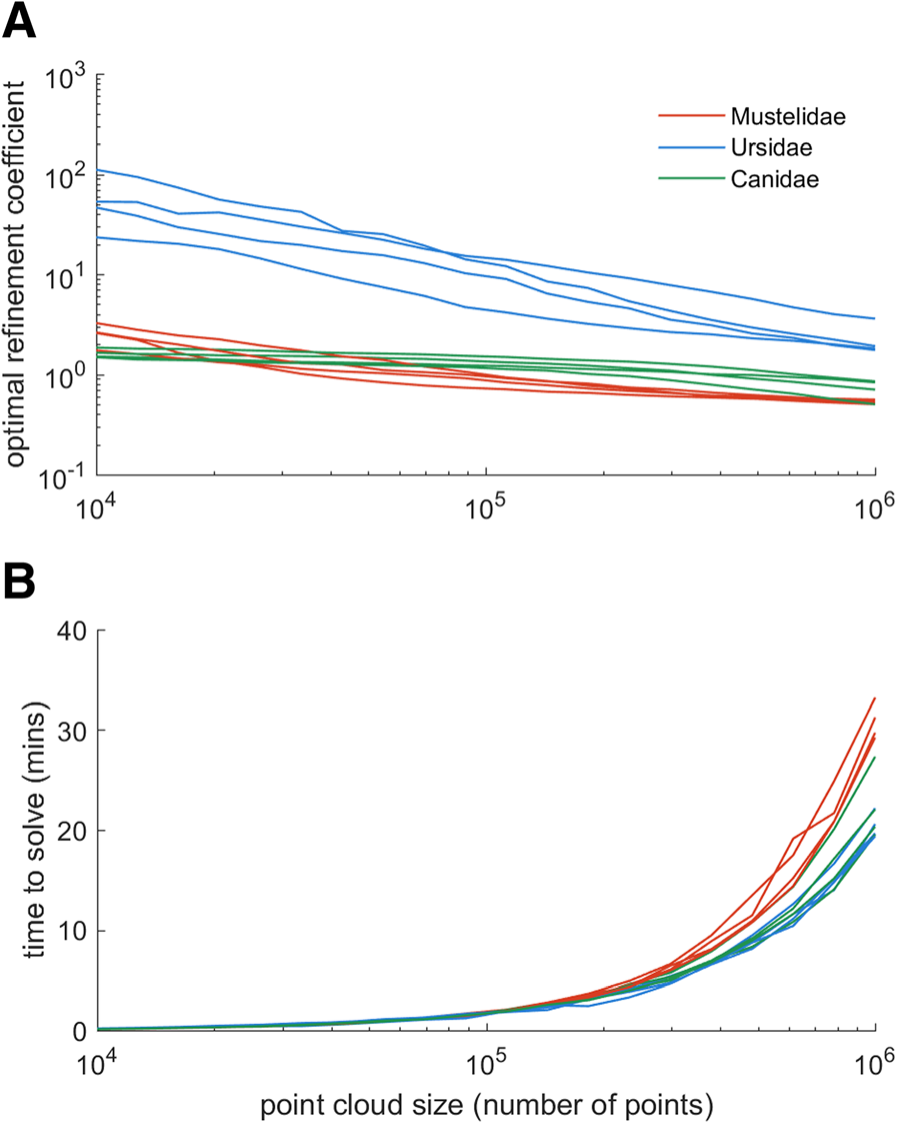
Alpha-shape sensitivity analysis. **a**, optimal refinement coefficients for study species over a range of point cloud densities. **b**, the associated computational time to find the optimum refinement coefficient for a given point cloud density

The results of the sensitivity analysis indicate that optimal refinement coefficients decrease with increasing point cloud size (Fig. 7a). Less dense point clouds require relatively coarser refinement coefficients in order to produce alpha shapes of equal volume to the original dataset. This phenomenon has previously been documented elsewhere, and has been referred to as the ‘coastline paradox’ ([75], see Discussion). Between 10^5^ and 10^6^ points, the rank order of optimal refinement coefficients remains relatively consistent across taxa (Fig. 7a). At the lowest point cloud densities, canids are considered the most ‘complex’, whilst mustelid bacula would appear most complex at point cloud densities of ~ 10^5^ points (Fig. 7a). This simply reflects the *scale* at which shape complexity is present. Canid bacula possess ‘gross’ complexity (e.g. presence of a deep urethral groove), whilst mustelid bacula are characterised by a more refined level of complexity (e.g. a shallow urethral groove, curved tip and complex apices) which may only be recovered at higher point cloud densities. The time taken to compute optimal refinement coefficients increases dramatically between 10^5^ points (1–2 min per specimen) and 10^6^ points (15–35 min per specimen) (Fig. 7b).

## Discussion

The alpha-shapes methodology presented here represents an additional tool for quantifying 3D shape complexity across biological samples characterised by high morphological disparity. Alpha-shapes operates by converting thresholded CT data directly to point clouds, thereby removing the requirement to surface mesh structures beforehand. The alpha-shapes algorithm does produce a suite of surface meshes as an output however, which may be incorporated into subsequent functional analyses. For example, the impact of the canid urethral groove on the biomechanical performance of the baculum may be quantified by constructing a suite of finite element models, based on coarser (groove absent) alpha-shapes and finer (groove present) alpha-shapes. The alpha-shapes algorithm is implemented in programming languages including MATLAB (‘alphaShape’) and R via the ‘alphahull’ package [68], thereby facilitating greater automatization in the future. Furthermore, alpha-shapes functionality is also present in the freeware software ‘Meshlab’ [54] for those preferring a graphical user interface.

A recent phylogenetic reconstruction of mammalian baculum presence/absence found support for the independent evolution of the structure on 8–9 occasions, with at least two independent gains of baculum within primates [76]. As alpha-shapes does not require the placement of homologous landmarks, it may therefore be extended to the analysis of potentially analogous structures or used to quantify shape complexity through ontogenetic sequences. We do also urge caution against the a priori assumption of analogous baculum function for mammals however, as no consistent relationship has yet been identified linking features of the baculum to underlying organismal biology across the whole group.

Here we find agreement between alpha-shapes, DI and DNE techniques in identifying ursid bacula as possessing low topographic complexity (Table 2, Fig. 6). This is perhaps unsurprising, as bear bacula lack both grooves/ridges/curvature *at a macro scale* and possess a smooth surface texture *on a finer scale*. In contrast, alpha shapes departs from the other complexity metrics in classifying mustelid bacula as more complex than canids, a pattern that is reversed in DI and DNE values (Table 2, Fig. 6). Disagreement between metrics of shape complexity is not unprecedented [22], and suggests the methods are simply capturing different aspects of complexity.

In this instance, we interpret these differences as being due to the relative sensitivity of each metric to concave versus convex topology. In DI and DNE, any change in topology (concave or convex) will contribute approximately equally to the complexity metric. In contrast, the calculated optimal alpha appears to be more influenced by the presence of concave sections. In Fig. 5, for example, alpha shapes fitted to the convex dorsal surface of the canid baculum change very little across two orders of magnitude in refinement coefficient. In contrast, the form of the alpha shape fitted to the highly concave ventral margin varies substantially alongside refinement coefficient. Thus, for specimens possessing large concave surfaces such as the urethral groove or distal tip curvature, small values of refinement coefficient are necessary for said features to be resolved. We consider alpha-shapes complexity to therefore be weighted more towards gross concave features than corrugated-like surface rugosity, in which convex and concave sections occur with approximately equal frequency and magnitude.

**Fig. 8:**
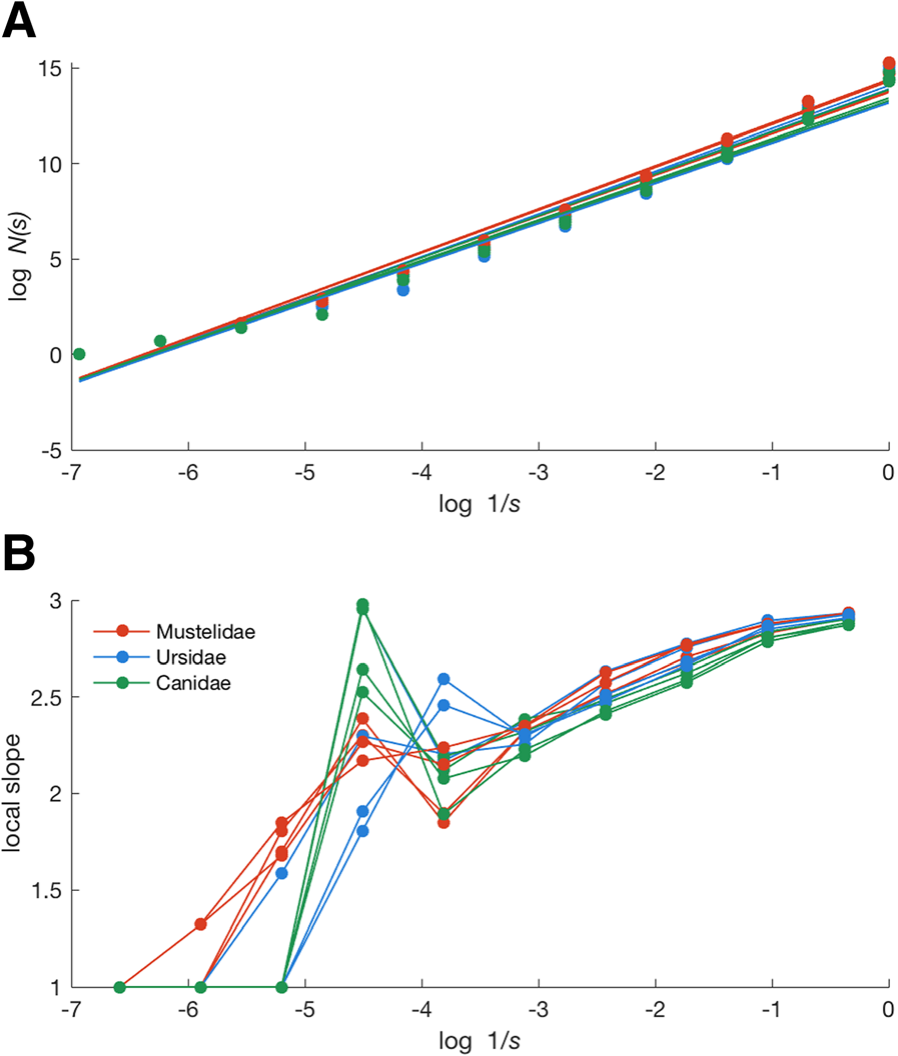
Box-counting estimation of fractal dimension (FD). **a**, Fractal dimension is calculated as the slope of the relationship between log(1/*s*) and log *N(s),* where *s* is the length of box edge and *N(s)* is the number of boxes required to cover the object. Steeper slopes are associated with increased topographical complexity; **b**, the local slopes as calculated between sequential data points of Fig. 8a. When objects are said to exhibit ‘true’ fractal behaviour, the local slope will plateau over a range of box sizes. In this instance, it is clear that no such plateaus occur, and thus bacula cannot be considered ‘fractal’

Relative to other metrics of topographic complexity considered here, fractal dimension does not distinguish between taxonomic groupings (Table 2, Fig. 6). Indeed, Fig. 8b would suggest carnivore bacula do not exhibit self-similarity, and the application of FD to this structure is not justified. In the box-counting technique applied here, true fractal behaviour would be identified by a ‘plateauing’ in local slope values across several scales of box-size [77]. As can be seen in Fig. 8b, no such plateaus exist, and bacula cannot be considered to behave in a fractal manner across several orders of magnitude scale.

Whilst the alpha-shapes method is not heavily user intensive, the process of shape-fitting can be computationally costly. To expedite the process, point clouds are downsampled. However, our sensitivity analysis does indicate that optimal refinement coefficients are a function of point cloud density (Fig. 7a). In denser point clouds, surface textural information (such as attachment scars, and small fossae) are preserved and a finer ‘fit’ around such features in necessary in order to recreate the original volume. At lower point cloud densities, only gross morphology is preserved and a coarser ‘optimal’ refinement coefficient is sufficient.

**Fig. 9:**
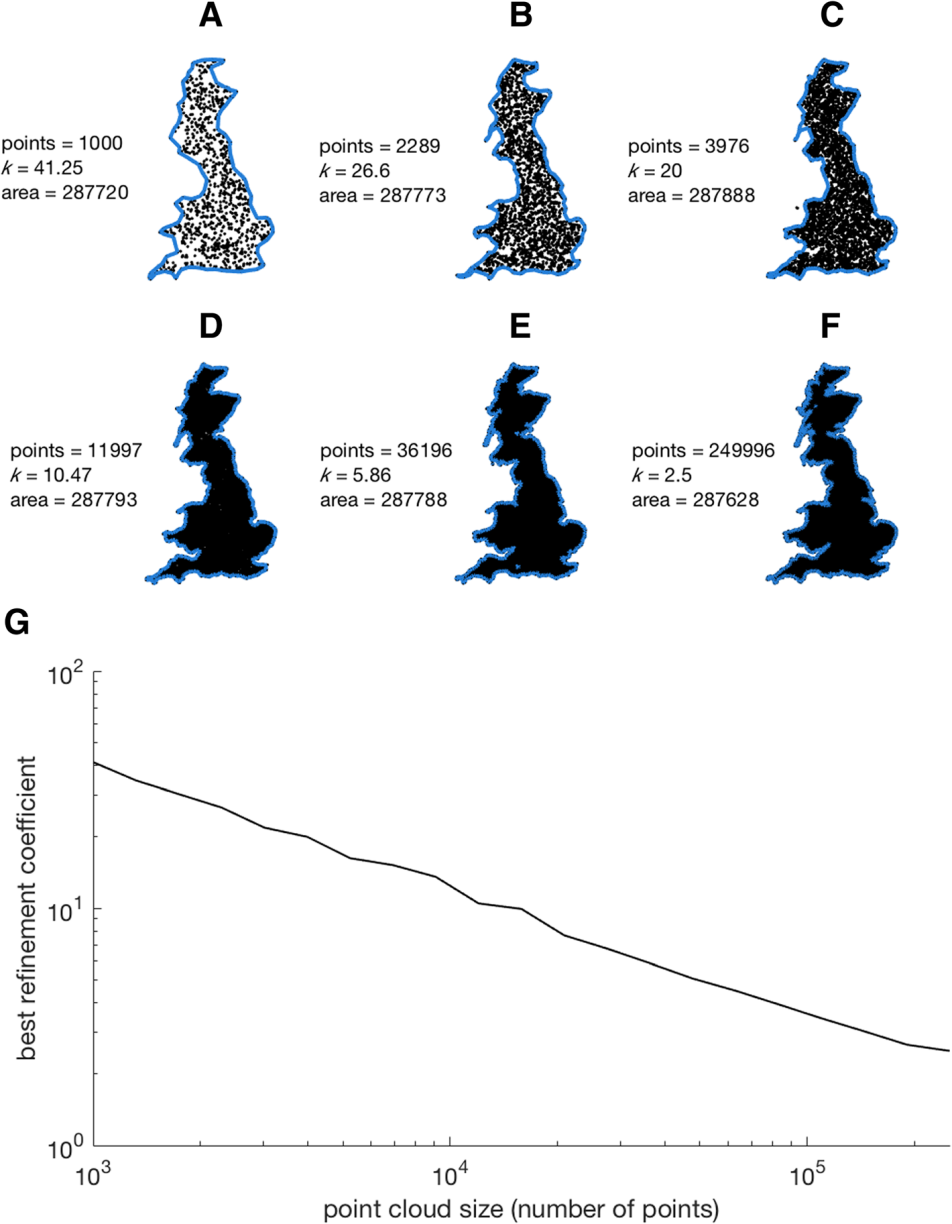
‘Coastline paradox’ example. 2D point clouds of Great Britain increase in density from **a**-**f**. As point cloud density increases, refinement coefficient *k* must decrease in order to resolve fine-scale features and maintain an equivalent alpha-shape area. Map modified from https://upload.wikimedia.org/wikipedia/commons/a/ab/England%2C_Scotland_and_Wales_within_the_UK_and_Europe.svg

This effect is related to a well know phenomena known as the ‘coastline paradox’ [65], in which the length of a country’s coastline increases as the scale of the measuring unit is decreased. Intuitively, more features of a coastline can be resolved and incorporated into a metric of length when using a shorter ‘measuring stick’. In the case of alpha-shapes, as point cloud density is downsampled, the likelihood of removing points lying on the outer contour is increased. As the outermost points define the margins of the specimen, downsampling results in an apparent ‘smoothing’ of the object and hence a coarser optimal refinement coefficient. To illustrate this effect, the alpha-shapes methodology was applied to a 2D point cloud map of Great Britain (Fig. 9). The results mirror our baculum dataset, with denser point cloud maps requiring more refined alpha-shapes in order to match the original area (Fig. 9). Low density point clouds loose many of the finer features of the coastline (thin peninsulas, bays etc.) and only gross shape is preserved.

Furthermore, the sensitivity analysis highlighted a change in the rank order of species’ optimal refinement coefficients associated with downsampling between 10^4^ and 10^5^ points (Fig. 7a). As discussed above, this pertains to the ‘hierarchy’ of complexity which may be revealed at a given point cloud size. Canids possess ‘gross’ complexity which may be resolved in low resolution point clouds, whilst mustelids are characterised by concave features of micro-complexity which require higher density point clouds to be detected. Beyond 10^5^ points, rank orders are relatively stable yet computational time increases dramatically (Fig. 6b).

Ultimately, point cloud density will be at the discretion of the user. This is not unusual, and similar decisions are made (implicitly or explicitly) whenever selecting the required resolution of a digital photograph or μCT scan. As a rule of thumb in μCT scanning, voxel size must be *at most* one-quarter to one-third of the size of the feature of interest in order to resolve said feature and avoid partial volume effects. Similarly, to guarantee their inclusion in an alpha-shapes analysis, we recommend the minimum dimension of a given feature (for example, the width of a groove or diameter of a fossae) comprise at least 3–4 data points within the point cloud. Beyond this, the final point cloud density will reflect a compromise between the level of detail required by the user and computer processing time.

That ‘optimal’ refinement coefficients are a function of point cloud density is not problematic for the application of alpha-shapes within a comparative analysis framework. Minimum point cloud density should be dictated by the smallest feature of interest *across the whole sample,* and all specimens downsampled to this same degree. Thus, ‘optimal’ refinement coefficients are equivalent across a given dataset. The *absolute* values of refinement coefficients will be specific to that given dataset however.

**Fig. 10:**
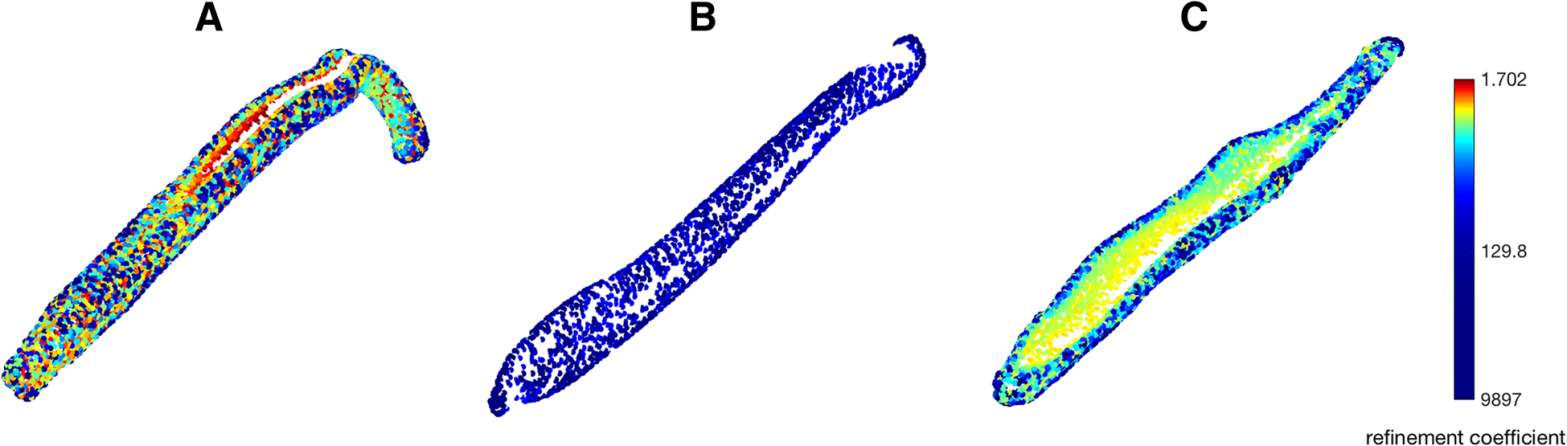
‘Coarsest refinement’ point clouds. Ventral surfaces of mustelid, ursid and canid bacula (left to right). Points are coloured according to the coarsest alpha-shape to which they contribute. The urethral groove of the mustelid baculum is identified as being particularly ‘complex’ according to the alpha-shapes methodology

In addition, the current implementation of alpha-shapes is limited in the sense that between-subject variation in alpha volume can be difficult to ascribe any one particular geometric feature. Figure 10 represents an initial attempt to address this shortcoming, in which data points of the point cloud are coloured according to the coarsest alpha-shape to which they contribute. The urethral groove of the canid requires a similar level of refinement in order to be resolved as the curved tip of the mustelid (Fig. 10, green), and would therefore be consider equally ‘complex’ in the current implementation of alpha—shapes. In contrast, the urethral groove of the mustelid required a more refined ‘fit’ of alpha in order to be distinguished (Fig. 10, red), contributing to the low values of optimal alpha calculated for all mustelids here. Future implementations of alpha-shapes will seek to further quantify regional variation in shape complexity within specimens, and will explore means of extracting additional information from alpha-curves.

## Conclusions

The alpha-shapes methodology presented here is an important addition to the biologist’s tool kit, providing a metric of topographical complexity that complements and extends pre-existing techniques such as Dissection Index, Fractal Index and Dirichlet Normal Energy. In particular, alpha-shapes appear sensitive to surface concavities, with features such as grooves and pits considered particularly ‘complex’. Alpha-shapes differs from methods that have previously been applied to genital shape, such as GMM and spherical harmonics, in that it describes the *extent* to which an object is structural complex, as opposed to how objects differ in the positioning of particular features. We therefore consider alpha-shapes to be especially useful for measuring the functional properties of shapes, be those animal genitals, corals, or the occlusal surfaces of teeth. Because optimal alpha values reflect the topographical complexity of a surface, rather than the specifics of how that complexity is achieved, it does not require the placement of homologous landmarks and may therefore be used to compare shape complexity across unrelated structures.

## Abbreviations

CT: Computed tomography
DI: Dissection index
DNE: Dirichlet normal energy
FD: Fractal dimension
GMM: Geometric morphometrics
LiDAR: Light detection and range
SPHARM: Spherical harmonics
